# Preoperative administration of local infiltration anaesthesia decreases perioperative blood loss during total knee arthroplasty – a randomised controlled trial

**DOI:** 10.1186/s40634-022-00552-1

**Published:** 2022-12-12

**Authors:** Oscar Lapidus, Mathilde Baekkevold, Pierre Rotzius, Lasse J. Lapidus, Karl Eriksson

**Affiliations:** 1 Department of Clinical Science and Education Södersjukhuset, Karolinska Institutet, Unit of Orthopaedics, Södersjukhuset, Sjukhusbacken 10, 118 83 Stockholm, Sweden; 2 Department of Orthopaedics, Södersjukhuset, Stockholm, Sweden

**Keywords:** Total knee arthroplasty (TKA), Local infiltration anaesthesia (LIA), Blood loss, Tourniquet, Pain, Osteoarthritis, Ropivacaine, Epinephrine, Ketorolac

## Abstract

**Purpose:**

Local infiltration anaesthesia (LIA) consisting of ropivacaine, epinephrine and ketorolac administered at the end of surgery has become the gold standard for postoperative analgesia as it provides improved postoperative pain relief compared to other methods. The use of LIA has retrospectively been shown to be associated with decreased perioperative blood loss. However, no randomised controlled trials have examined the effect of of preoperative LIA on blood loss. This study aimed to compare pre- vs perioperative LIA during TKA surgery, with a primary outcome of perioperative blood loss.

**Methods:**

The present study was performed as a prospective single-center randomised controlled trial. A total of 100 patients undergoing primary TKA between October 2016 and March 2018 were randomised to receive either pre- or perioperative LIA. Perioperative blood loss was measured, as well as pre- and postoperative haemoglobin levels. Postoperative pain was estimated at intervals approximately 24, 48 and 72 hours after surgery; analgesic drug consumption was recorded for each patient, as well as the total length of stay as an in-patient.

**Results:**

Ninety six patients received either pre- or perioperative LIA as part of the intervention and control group respectively. Average blood loss was 39% lower in the intervention group at 130 ml vs 212 ml in the control group (*p*=0.002). No significant difference in haemoglobin drop, postoperative pain or length of hospital stay was found.

**Conclusions:**

Preoperative LIA resulted in a 39% decrease in perioperative blood loss during TKA surgery compared to perioperative administration while providing non-inferior postoperative pain relief.

## Background

In orthopaedic surgery such as total knee arthroplasty (TKA), haemostatic control is essential not only to optimise visualisation but also to reduce postoperative swelling, allowing for adequate range of motion and early mobilization [1–3]. Tourniquet use during TKA surgery has decreased in recent years in part due to risks associated with prolonged haemostasis, but also because studies have shown that postoperative blood loss is increased when compared to surgery performed without tourniquet [4, 5]. Tourniquet-free surgery is non-inferior regarding cement penetration and prosthesis fixation as well as associated with a lower risk of infection, but studies report mixed results of reduction in perioperative blood loss [4–6]. Due to decreasing use of perioperative tourniquets and the consequent risk of increased blood loss, alternative methods of reducing perioperative blood loss are needed to optimise visualisation during surgery.

Local infiltration anaesthesia (LIA) has become the gold standard for postoperative analgesia in TKA surgery as it provides improved postoperative pain relief and allows for earlier mobilisation than other methods [1, 3, 7]. During surgery it is also possible to leave an embedded subcutaneous catheter for postoperative administration of additional local anaesthetic drugs, although this is associated with a greater risk of infection [8, 9]. Due to its vasoactive effects, LIA consisting of ropivacaine, epinephrine and ketorolac can potentially be utilised to decrease perioperative blood loss if administered before the start of surgery. One retrospective study found a significant correlation between the use of LIA and reduced blood loss during TKA surgery by estimating haemoglobin dilution, but no randomised trials have examined the effect of preoperative LIA on blood loss [10]. This study therefore aimed to compare pre- vs perioperative LIA during TKA surgery, with a primary outcome of perioperative blood loss. Secondary outcomes were duration of surgery, postoperative self-reported pain, consumption of analgesic drugs as well as total length of in-patients hospital stay. The hypothesis was that preoperative LIA reduces perioperative blood loss while also providing adequate postoperative pain relief.

## Methods

This study was performed as a single-center randomised controlled trial, in which a total of 100 patients undergoing primary TKA between October 2016 and March 2018 at Södersjukhuset, Sweden were included in this study. Inclusion criteria were 40-90 years of age, no known allergies to the drugs used in the intervention and control group. Exclusion criteria were current anticoagulant therapy. Surgery was performed by one of twelve surgeons involved in the study, all experienced in joint replacement surgery.

Patients were randomised using sealed envelopes administered by an independent research nurse to receive either pre- or perioperative LIA as part of the intervention and control group respectively. Both groups received LIA consisting of 100 ml of ropivacaine (2 mg/ml), 0.5 ml of epinephrine (1 mg/ml) and 1 ml of ketorolac (30 mg/ml). Preoperative LIA was given after spinal block/general anaesthesia as a 50 ml injection around the joint capsule as well as 50 ml intraarticularly 10-20 minutes before the start of surgery. Perioperative LIA was given as a 100 ml injection around the joint capsule during surgery at the end of surgery. Both groups also received 50 ml of periarticular ropivacaine (50 mg/ml) at the end of surgery immediately before wound closure. 10 ml of tranexamic acid (100 mg/ml) was administered intravenously twice during each surgery (immediately preoperatively and before wound closure) in both groups to reduce blood loss [11–13].

Perioperative blood loss was estimated and recorded by the surgical staff by measuring the weight of saturated surgical cloths and the total suction retrieval, then subtracting the weight of the dry cloth as well as the total amount of irrigation fluid used. Pre- and postoperative haemoglobin levels were also recorded, as well as the total surgical time. Patients were asked to estimate their current level of pain according to the Numeric Pain Rating Scale (between 0 and 10) at intervals approximately 24, 48 and 72 hours after surgery; consumption of analgesic drugs was recorded for each patient, as well as the total length of stay as an in-patient.

Statistical analysis was performed with a significance level of 0.05. Shapiro-Wilk tests were used to determine which variables had normal distribution. Independent samples T-test was used to compare normally distributed continuous data. Non-parametric tests such as the Mann-Whitney U test and Chi-squared test were used to compare non-normally distributed continuous and categorical data respectively. Mean values and standard deviation were determined for normally distributed data; median values and interquartile range were reported for variables with non-normal distribution (Table 1). Prior to initiation, a clinical pilot trial was performed, after which a power analysis revealed the need for 25 patients in each group to accurately evaluate the intervention protocol.

**Table 1:** Comparison of intervention and control group

	Preoperative LIA	Perioperative LIA	Significance
Number of patients	47 (49.0%)	49 (51.0%)	
Age			
mean (SD)	69.8 (11.1)	67.6 (9.3)	
median (range)	71 (46-91 )	66 (50-88)	
Sex			
male	17 (44%)	22 (56%)	
female	30 (53%)	27 (47%)	
BMI (kg/m2)			
mean (SD)	29.8 (5.0)	28.4 (5.3)	
Blood loss (ml)			
mean (SD)	130 (118)	212 (123)	0.002
median (IQR)	100 (100)	183 (200)	<0.001
not reported	1 (2.1%)	5 (10.2%)	
Haemoglobin drop (g/L)			
mean (SD)	25 (10)	28 (11)	0.24
not reported	4 (8.5%)	6 (12.3%)	
Duration of surgery (minutes)			
median (IQR)	73 (26.5)	80 (24.5)	0.11
not reported	2 (4.3%)	1 (2.0%)	
Self-reported pain (NRS)			
24 hours, mean (SD)	5.0 (1.9)	4.9 (1.7)	0.92
not reported	3 (6.4%)	0 (0%)	
48 hours, mean (SD)	4.2 (1.7)	3.9 (1.7)	0.40
not reported	10 (21.3%)	8 (16.3%)	
day 1 & 2, mean (SD)	4.7 (1.6)	4.4 (1.6)	0.34
not reported	10 (21.3%)	8 (16.3%)	
Time in hospital			
median (IQR)	3 (1)	2 (1)	0.19
not reported	1 (2.1%)	0 (0%)	

## Results

A total of 50 patients were randomised to be part of the intervention group, as well as 50 to comprise the control group. Four patients were excluded by the surgeon after randomisation for the following reasons: allergy (*n*=1), logistic reasons (*n*=1) and protocol violation (*n*=2). Of the remaining 96 patients, 47 patients received preoperative LIA as part of the intervention group (17 males, 30 females), whereas 49 patients received perioperative LIA (22 males, 27 females); mean age at the time of surgery was 69.8 and 67.6 years in the intervention- and control groups respectively (Table 1).

Average blood loss was 39% lower in the intervention group with a mean of 130 (±118) ml vs 212 (±123) ml in the control group (*p*=0.002); median values were 100 ml and 183 ml respectively (*p*<0.001) (Table 1). No patients required transfusion of blood products. There was a trend towards shorter duration of surgery in the group that received preoperative LIA (77.4 vs 84.4 minutes), although this trend was not statistically significant (*p*=0.11). No significant difference was found in perioperative haemoglobin drop (*p*=0.24), self-reported postoperative pain (*p*=0.92) or total length of in-patient hospital stay (*p*=0.19) (Table 1). Consumption of analgesic drugs could not accurately be measured due to patients being discharged earlier than expected. No surgical or immediate postoperative complications were reported for any patient during the hospital stay.

## Discussion

This study found a 39% decrease in perioperative blood loss during TKA surgery following preoperative administration of LIA compared to perioperative administration. No studies examining blood loss during TKA surgery with different protocols of LIA were available for comparison.

In this study, blood loss was measured by weighing saturated surgical cloths and measuring irrigation fluid to objectively measure blood loss, as opposed to estimation of blood loss through the use of a formula. Limitations include a possible methodical error in measurement of blood loss by weighing saturated surgical cloths, as blood absorbed by surgical drapes and lost on the floor was either not recorded or estimated by the surgical staff. However, this is likely only a fraction of the total blood loss, and the effects should therefore be similar in the intervention and control group. This is also compensated by the large sample size of this study. Estimating perioperative blood loss is difficult and this method was chosen to objectively quantify blood loss by means other than estimation. As the total blood loss is rarely large enough to significantly impact haemoglobin levels, estimating blood loss by calculating hemoglobin dilution was deemed unsuitable. Although this measurement may arguably not reflect the true blood loss of each patient, the margin of error should be approximately equal in all cases.

Tranexamic acid was administered intravenously twice during surgery for patients in both groups to reduce blood loss. While this may have decreased the total blood loss, the effects would be equal in both groups. It is possible that administration of a higher dose would have further reduced blood loss in both groups, rendering the effects of perioperative LIA non-significant. However, the use of tranexamic acid is not without risk, and preoperative LIA has the advantage of not only reducing blood loss but also providing postoperative pain relief.

No significant difference in self-reported pain 24 hours after surgery was found, hence this study suggests that preoperative LIA does not provide inferior postoperative pain relief compared to perioperative administration. All patients were given postoperative oral analgesic drugs (paracetamol as well as short- and long-acting oxycodone) according to the same protocol. Unfortunately, consumption could not accurately be assessed, in part because 18 patients were discharged during the first 24 hours after surgery. There are also various psychological factors affecting tendency to consume postoperative oral analgesic drugs, which this study was not designed to adjust for.

The decision of involving twelve different surgeons performing TKA was done to accurately mimic the clinical setting and for the results to be clinically relevant to the department. Although this may influence the results for any given patient, the randomized study design, as well as having a sample size in far excess of what was deemed necessary by the power analysis, should suffice for these differences to be negligible when comparing the intervention and control group. This also reflects the clinical reality of many emergency hospitals, and the results should therefore be applicable at other similar orthopaedic units and allow for a high degree of replicability.

Although this study demonstrates a decrease in perioperative blood loss, the clinical relevance of this finding is debatable as the mean total blood loss in both groups was 130 ml and 212 ml respectively. However, the trend towards shorter duration of surgery in the intervention group could potentially be due to less blood in the surgical field allowing for improved visualisation and shorter surgical time.

## Conclusion

Preoperative administration of LIA (ropivacaine, ketorolac and epinephrine) resulted in a 39% decrease in perioperative blood loss during TKA surgery and provided non-inferior postoperative pain relief compared to perioperative administration. This finding supports the use of early administration of LIA and emphasises the timing of administration as decreased blood loss is desired to ensure adequate visualisation during surgery as well as to allow for early postoperative mobilisation.

## Data Availability

The data will not be published but is available from the corresponding author at reasonable request.

## Abbreviations

TKA: Total knee arthroplasty
LIA: Local infiltration anaesthesia
KOOS: Knee injury and osteoarthritis outcome score
NRS: Numeric pain rating scale
BMI: Body mass index
